# Determinants of persistence in hypertensive patients treated with irbesartan: results of a postmarketing survey

**DOI:** 10.1186/1471-2261-5-13

**Published:** 2005-06-08

**Authors:** Michel Burnier, Bernhard Hess, Peter Greminger, Bernard Waeber

**Affiliations:** 1 Service de Néphrologie, CHUV, Lausanne, Switzerland; 2 Spital Zimmerberg, Wädenswil, Switzerland; 3 Medizinische Poliklinik, Universitätsspital, Zürich, Switzerland; 4 Division de Physiopathologie Clinique, CHUV, Lausanne, Switzerland

## Abstract

**Background:**

Persistence is a key factor for long-term blood pressure control, which is of high prognostic importance for patients at increased cardiovascular risk. Here we present the results of a post-marketing survey including 4769 hypertensive patients treated with irbesartan in 886 general practices in Switzerland. The goal of this survey was to evaluate the tolerance and the blood pressure lowering effect of irbesartan as well as the factors affecting persistence in a large unselected population.

**Methods:**

Prospective observational survey conducted in general practices in all regions of Switzerland. Previously untreated and uncontrolled pre-treated patients were started with a daily dose of 150 mg irbesartan and followed up to 6 months.

**Results:**

After an observation time slightly exceeding 4 months, the average reduction in systolic and diastolic blood pressure was 20 (95% confidence interval (CI) -19.6 to -20.7 mmHg) and 12 mmHg (95% CI -11.4 to -12.1 mmHg), respectively. At this time, 26% of patients had a blood pressure < 140/90 mmHg and 60% had a diastolic blood pressure < 90 mmHg. The drug was well tolerated with an incidence of adverse events (dizziness, headaches,...) of 8.0%. In this survey more than 80% of patients were still on irbesartan at 4 month. The most important factors predictive of persistence were the tolerability profile and the ability to achieve a blood pressure target ≤ 140/90 mmHg before visit 2. Patients who switched from a fixed combination treatment tended to discontinue irbesartan more often whereas those who abandoned the previous treatment because of cough (a class side effect of ACE-Inhibitors) were more persistent with irbesartan.

**Conclusion:**

The results of this survey confirm that irbesartan is effective, well tolerated and well accepted by patients, as indicated by the good persistence. This post-marketing survey also emphasizes the importance of the tolerability profile and of achieving an early control of blood pressure as positive predictors of persistence.

## Background

Angiotensin II receptor antagonists (AIIRA) have demonstrated excellent efficacy in patients with hypertension [1,2], heart failure [1,2], diabetes [3], diabetic nephropathy [4-6], and recently in post-myocardial infarction patients [7]. Such promising results allowed the US Joint National Committee on hypertension in their seventh report (JNC-7) and the European Society of Hypertension (ESH-ESC) in the 2003 guidelines to integrate this class of agents in the management of hypertension and to propose AIIRAs as an alternative treatment to ACE-inhibitors for most of the above-mentioned high-risk conditions [8,9].

However, the main message of the published guidelines remains that a normalization of arterial blood pressure in hypertensive patients is the key objective to the prevention of cardiovascular events, especially in high-risk categories, where stricter therapeutic targets and aggressive strategies to reach them have been proposed. Yet, achieving normalization of blood pressure (BP) remains a difficult task, or, in the words of the JNC-7 report [8]: "The most effective therapy prescribed by the most careful clinician will control hypertension only if the patient is motivated to take the prescribed medication and to establish and maintain a health-promoting lifestyle." In industrialized countries, the success rates in controlling blood pressure range from below 10% to 30% of the treated population, depending mainly on the definition of the therapeutic targets [10-14]. The JNC-7 report estimates that 34% of hypertensive patients in the USA manage to maintain a BP below 140/90 mmHg [8].

The inadequate persistence with therapy, i.e. the frequent switch or discontinuation of the prescribed therapy has been recognized as a frequent cause of treatment failure [15]. Persistence is a good general indicator of the satisfaction with the treatment of both patients and physicians. Among classes, a British comparative study showed ACE-inhibitors as the best and diuretics as the poorest persistence builders [15]. A large Canadian cohort study based on pharmacy prescriptions confirmed these results [16]. Recently, the first population-based studies including AIIRAs revealed that patients persist more with AIIRAs than with all other antihypertensive drugs after 6 months up to 3 years [17,18]. A cohort study with 2416 newly diagnosed hypertensive patients showed that the AIIRA irbesartan induced significantly more persistence than other drug classes and even than other AIIRAs [19]. This improved persistence has been attributed in part to the efficacy of the compounds and mainly to the low, placebo-like side effects profile [20-22]. Placebo-like tolerability has indeed been confirmed at all clinically relevant dosages of irbesartan [23].

The aim of this open prospective observational survey with 4769 hypertensive patients treated in general practices in Switzerland, was to evaluate the persistence with irbesartan in real-life settings over a period of about one year and to investigate factors affecting either positively or negatively the persistence. In addition, the tolerability profile and the effect of irbesartan on BP control were assessed.

## Methods

### Design of the investigation

This prospective observational survey was conducted in general practices in all regions of Switzerland from October 1997 to March 1999 i.e. shortly after the launch of irbesartan in the country. The general practitioners (GPs) were asked to document their daily routine in the treatment of hypertensive patients with irbesartan; 1390 physicians were contacted, 1045 included patients and 886 documented the treatment. They were recruited by the field forces of the sponsors of the study i.e. BMS and Sanofi-Synthelabo Switzerland. GPs filled in a baseline visit (Visit 1) form for every treated patient, and could report up to five follow up visits in the following 3–6 months (End Form). They were asked to document two arterial blood pressure measurements at every visit and to report concomitant antihypertensive medication and adverse events, as well as changes or discontinuation of treatment. At the end of the observation period, GPs reported if their patients were continuing the treatment with irbesartan, alone or in combination with other antihypertensive drugs. About 1 year after baseline, patients with ongoing treatment with irbesartan received a Compliance Form Patient, where they were asked how irbesartan was tolerated (very well, well, fairly well, poorly) and how many times weekly they took irbesartan in the preceding 3–4 weeks; a further question was how many tablets (all drugs) were taken daily. Their GPs received a Compliance Form MD, to report the last blood pressure measurement and if and when the patient had discontinued the treatment. The various forms were designed by external consultants. The data were collected by mail using self-addressed envelopes and processed by the consultants. Queries for incomplete forms were done by the sponsor representatives. No standard validated questionnaire was used. In Switzerland, this type of survey does not need to be submitted to an ethical committee.

### Patient selection

All patients with newly diagnosed hypertension, or with treated hypertension requiring a change in medication according to the GP, were considered for the survey. No standardized definition was used but physicians considered a BP >140/90 mmHg as hypertension. There were neither demographic nor clinical exclusion criteria. The only condition to participate was that patients should not have been pre-treated with irbesartan. Treatment was started with irbesartan 150 qd. Thereafter, physicians were free to change the antihypertensive therapy at any time during the follow-up based on their individual therapeutic goals (usually <140/90 mmHg).

### Statistical analysis

Values are presented as mean +/- sd. The statistical significance of between-group differences was computed using the 2-sided Chi-square test, the Mann-Whitney-U test or ANOVA methods as appropriate. For multivariate correlations, a logistic regression analysis with a dichotomous dependent variable was used (e.g. therapy discontinuation: yes = 1; no = 0). To support the results of the logistic regression models, Cox regression models with cumulative survival functions were further computed.

## Results

### Characteristics of the database

**Figure 1:**
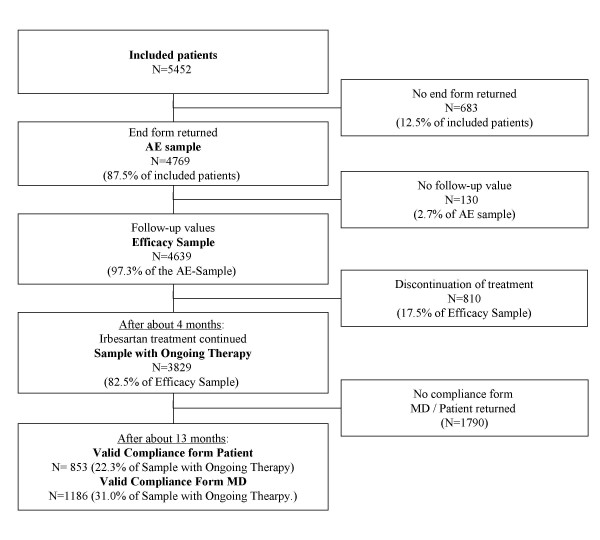
**Patient population and available data. **Summary of the analysis patient populations of this investigation and of the data available for analysis.

As shown in Figure 1, 5452 patients were enrolled by 1045 GPs, and 886 of those returned the therapy documentation (End Form) of 4769 patients (87.5%). This latter sample was taken to analyze the safety data and is referred to as AE-Sample (Tolerability Events). For the evaluation of the effect on BP control, all cases with at least one follow-up value were taken into account. 130 cases of the AE-Sample had no follow-up values; the remaining 4639 subjects (97.3% of the AE-Sample) are referred to as the Efficacy Sample. At the end of the treatment observation period, after an average of slightly more than 4 months (133 ± 75 days, mean ± SD), GPs reported that 3829 patients (82.5% of the Efficacy sample) continued the treatment with irbesartan. This is referred to as the Sample with Ongoing Therapy. A total of 1419 Compliance Forms MD (37.1% of the sample with ongoing therapy) and 928 Compliance Forms Patient (24.2%) were returned after on average more than 13 months from baseline (402 ± 105 days). Due to lack of completeness, some forms had to be excluded from the analysis, giving a total of 1186 valid Compliance Forms MD (31.0%) and 853 cases with both usable Compliance Form MD and Patient (22.2%).

Table 1 summarizes the baseline demographic and clinical data for both the previously untreated and the pre-treated patients. Almost two thirds of the patients (61,5%) entered the study receiving another therapy for high blood pressure. The most frequent reasons why GPs changed pre-treatment to introduce irbesartan were insufficient efficacy of the previous therapy (64.6%), cough (22.5%) and adverse events other than cough (16.6%). The multivariate analysis of factors correlated to pre-treatment shows that patients who switched to irbesartan from other antihypertensive drugs were older, prevalently female and from the German part of Switzerland (p < 0.001). They had significantly more risk factors, associated clinical conditions (p < 0.0001) and target organ damages than naïve patients (p = 0.0013). More pre-treated patients received a polytherapy regimen during the post-marketing surveillance (p < 0.0001).

**Table 1:** Baseline demographic and clinical data: Efficacy Sample.

	***Naïve patients***	***Pre-treated***	***Total***
Total	1785 (38.5%)	2854 (61.5%)	4639
Gender (f/m)	882/903	1605/1249	2487/2152
Mean age ± SD	57.9 ± 12.7	63.6 ± 12.3	61.4 ± 12.8
Baseline SBP (Mean ± SD)	168.8 ± 17.8	163.5 ± 19.1	165.5 ± 18.8
Baseline DBP (Mean ± SD)	101.2 ± 8.6	96.3 ± 10.4	98.2 ± 10.0
Diabetes	165 (9.2%)	492 (17.2%)	657 (14.2%)
ISH	79 (4.4%)	408 (14.3%)	487 (10.5%)
WHO-risk			
*Low (<15%)*	37 (2.1%)	101 (3.5%)	138 (3.0%)
*Medium (15–20%)*	837 (46.9%)	1190 (41.7%)	2027 (43.7%)
*High (20–30%)*	274 (15.4%)	526 (18.4%)	800 (17.2%)
*Very high (>30%)*	637 (35.7%)	1037 (36.3%)	1674 (36.1%)

For detailed explanation of WHO-risk and region categories see results. ISH = isolated systolic hypertension (defined as ≥ 140 mmHg systolic and < 90 mmHg diastolic blood pressure).

### Effect on blood pressure

**Figure 2:**
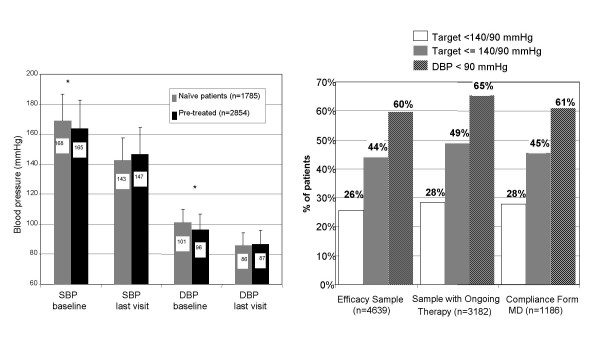
**a. Evolution of blood pressure during observation period (~4 months): Efficacy Sample**. Baseline SBP (systolic blood pressure): previously untreated patients 168.8 mmHg, pre-treated 163.5 mmHg (*p < 0.0001); SBP at last visit: previously untreated 142.8 mmHg, pre-treated 146.9 mmHg (p < 0.0001). Baseline DBP (diastolic blood pressure): previously untreated patients 101.2 mmHg, pre-treated 96.3 mmHg (*p < 0.0001); DBP at last visit: previously untreated 85.9 mmHg, pre-treated 86.8 mmHg (p = 0.004). **b. Reaching of therapeutic targets: Efficacy Sample. **Response to treatment is defined as reaching DBP < 90 mmHg or a reduction of DBP = 10 mmHg. In real-life practice, a satisfactory objective is also the normalization of DBP (< 90 mmHg). Target = 140/90 mmHg was introduced because of digit preference of study GPs (see results).

More that 90% of the Efficacy Sample patients received irbesartan 150 mg qd, as a monotherapy or in combination with other antihypertensives. 69% of the Efficacy Sample patients received a constant monotherapy and 5.6% a constant polytherapy. In the Efficacy Sample, the mean reduction of systolic blood pressure (SBP) from baseline to the last visit was 20.2 ± 19.5 mmHg (p < 0.001). For the diastolic blood pressure (DBP), the mean reduction was 11.7 ± 11.3 mmHg (p < 0.001). Figure 2a shows the differences between naïve and pre-treated patients. Despite previous treatment, the pre-treated group had clearly inadequate mean baseline values of SBP and DBP (163.5 and 96.3 mmHg, respectively) justifying a change in therapy. Naïve patients achieved a significantly greater reduction of both SBP and DBP than pre-treated ones. At the last visit, the pre-treated group showed higher SBP and similar DBP values in comparison to naïve patients.

Since GPs did not receive specific instructions about therapeutic goals in this survey, various therapeutic targets were used to evaluate the success in controlling blood pressure (Figure 2b). As shown in the figure, one third of the patients had normalized their BP (<140/90 mmHg) and two-thirds had a diastolic BP below 90 mmHg. The Sample with Ongoing Therapy, as reasonably expected, appeared slightly more successful than the Efficacy Sample in achieving the various targets (Figure 2b).

### Tolerability profile

Adverse events were reported for 383 patients (8.0% of the AE-Sample), more often by older patients (>65 years: 10.2%; 55–65 years: 7.8%; = 55 years: 5.5%; p < 0.001) and by pre-treated patients (9.6% vs. 5.5% naïve; p < 0.001). Yet, in the majority of patients (90.7%), irbesartan was well tolerated according to GPs. Tolerance was reported as poor only for 131 patients (2.7%). Adverse events led to discontinuation of irbesartan in 343 cases (7.4%). The most frequent side effects are listed in Table 2, where they are compared with their occurrence listed in the Swiss prescribing information [24]. Serious adverse events, leading to death, disability, life-threatening conditions or hospitalization, were reported in 74 patients (1,3% of AE-sample), but GPs described a possible or probable connection with trial medication only in 8 cases. Very good or good tolerance was reported by 824 patients in the Compliance Form Patient (96.6% of the total), all subgroups scoring above 90%.

**Table 2:** Most reported adverse events in the AE-Sample (n = 4769) compared to the Swiss prescribing guidance

	*AE-Sample*	*Swiss prescribing guidance*
**Total**	**383 (8.0%)**	
Dizziness	65 (1.4%)	>1%
Nausea	53 (1.1%)	>1%
Headache	43 (0.9%)	>1%
Dyspepsia	24 (0.5%)	0.5–1%
Diarrhea	18 (0.4%)	0.5–1%
Palpitation	17 (0.4%)	Not mentioned
Cough	15 (0.3%)	0.5–1%
Fatigue	15 (0.3%)	>1%
Vomiting	11 (0.2%)	>1%
Tachycardia	10 (0.2%)	0.5–1%

### Persistence

**Figure 3:**
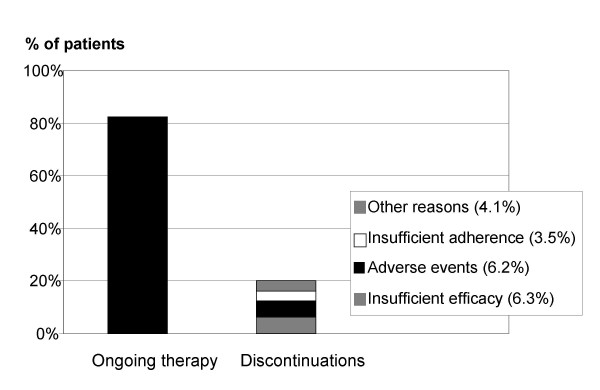
**Ongoing treatment and discontinuation reasons: **Efficacy Sample. (n = 4639)Other reasons included: patient moved, blood pressure normalized, break off attempt, concurrent disease, effect too strong, lost to follow up and others. Multiple answers were possible.

**Figure 4:**
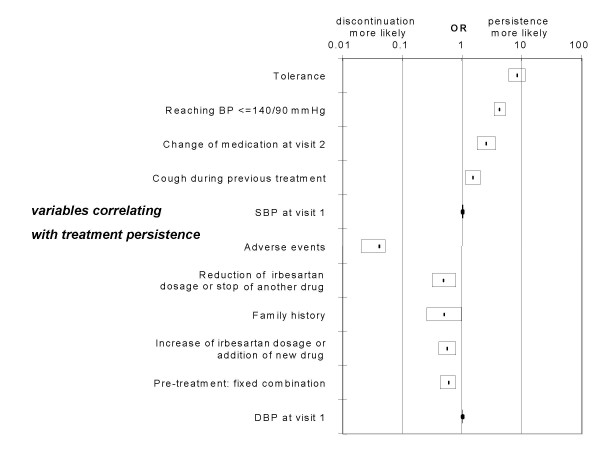
Mean (line) and 95% confidence interval (box) for the odds ratio (OR) of the main variables correlating significantly with ongoing treatment or discontinuation in a logistic regression model; Efficacy Sample. For detailed explanations see results.

3829 patients out of 4639 continued the treatment with irbesartan after the last visit (on average, more than 4 months from the start). The main reasons for discontinuation of the remaining 810 patients were the occurrence of adverse events and an insufficient efficacy (figure 3). In a logistic regression model, the factors that correlated more strongly with ongoing therapy were a reported good tolerance and reaching the a blood pressure ≤ 140/90 mmHg. Interestingly, pre-treated patients discontinued irbesartan significantly more often when the previous therapy was a fixed combination of antihypertensive agents, but not if they had stopped the pre-treatment because of cough, which on the contrary increased the probability of therapy continuation. Treatment modifications also affected persistence. Thus, if the dose of irbesartan was increased or another antihypertensive agent was added at the first follow up visit (Visit 2), patients had better chances to stay on therapy whereas, if treatment was modified after visit 2, this was associated with more discontinuations. In the former situation 9.5% of patients persisted on therapy. In contrast, if further changes in drug therapy were necessary on visit 3 because of insufficient BP control 35.9% of drug therapies were discontinued. The survival analysis (Cox regression) generally confirmed the statistically significant relationships with persistence found in the logistic regression models (Figure 4). An effect detected only by the Cox regression was the positive correlation between persistence and number of risk factors for cardiovascular diseases at baseline (RR = 0.74 for the discontinuation; p = 0.0001). Diabetes did not appear to influence persistence. In both multivariate analyses, the presence of a family history of hypertension or cardiovascular diseases reduced the chances of persistence.

### Perception of compliance

**Figure 5:**
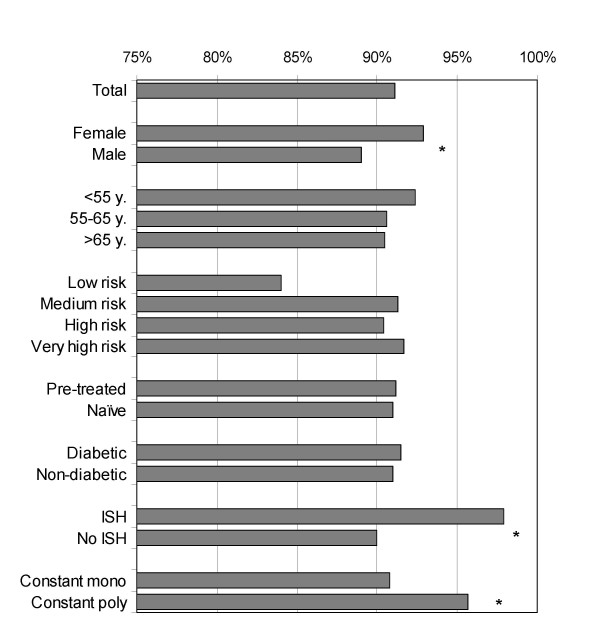
Self-reported compliance according to the patient for selected patient subgroups. Good compliance with treatment after 1 year; Compliance Form Patient (n = 853). Good compliance is defined as >80% adherence to the prescribed therapeutic regimen. In this case it means irbesartan intake on 6 or 7 days a week, as reported by the patients. Risk = WHO risk categories; ISH = isolated systolic hypertension; constant mono-and polytherapy; on & off = on and off treatment breaks; * = p < 0.05 compared to the rest of the patients.

Patients with ongoing therapy at last visit were asked in the Compliance Form Patient to indicate how many irbesartan tablets they took per week during the preceding 3–4 weeks. Since after about one year only 853 patients returned the form, we have to assume a sampling bias. Nevertheless, some within-group differences are worth mentioning. 777 patients (91.1%) who returned the Compliance Form Patient reported an irbesartan intake of 6–7 times per week, i.e. more than 80% of the prescribed doses. All subgroups scored around 90% (Figure 5), but females reported a better compliance than males (92.9% vs. 89.0%; p = 0.021). Patients with isolated systolic hypertension appear to adhere better to therapy than other hypertensives (97.2% vs. 90.2%; p = 0.032), while patients with a low risk for cardiovascular events showed a lower compliance (84%, n.s.). Compliance Forms MD reported an overall ongoing treatment rate with irbesartan of 88.0% (1044 patients), and a slightly higher rate for pre-treated patients (90.4%, n = 728).

## Discussion

Taken together, the data of this postmarketing survey confirm that irbesartan is a well tolerated and effective antihypertensive agent when used in a real life setting at the dose of 150 mg qd. More interestingly, our data provide further insights on factors affecting either positively or negatively the persistence with antihypertensive treatment. In particular, our observations point out the importance of a good tolerability profile and of achieving a rapid control of blood pressure in enhancing persistence whereas late changes in treatment and addition of irbesartan in patients already treated with a fixed-dose combination appears to be factors promoting a lower persistence.

Prospective observational surveys are not specifically designed to evaluate the antihypertensive efficacy of a new agent since there is generally no control group and the treatment schedule is not standardized. Moreover, there may be a selection bias since the inclusion of patients was not randomised. Nevertheless, this type of survey may provide some valid information on the antihypertensive effect that may be obtained in real life conditions i.e. outside the rigorous context of a clinical trial and the rather large number of patients included certainly limit the effect of a systematic selection bias. In the present case most patients were treated with a 150 mg irbesartan tablet per day because this was the dose recommended at the time the survey was conducted, i.e. soon after the launch of irbesartan in Switzerland. All patient subgroups, but in particular naïve patients, responded positively to the treatment. In fact, in the group of patients with follow-up values and after a mean observation time of 4 months, the average reductions in blood pressure were of comparable magnitude that those obtained in clinical trials and in other post-marketing surveys [25-27]. Such a substantial reduction could be achieved in spite of the fact that irbesartan 150 mg represents the minimal recommended daily dosage nowadays.

Of note, no blood pressure target was pre-defined in our program. Moreover, one should consider that the reported blood pressure values show a clear digit preference for figures ending with a 0 or a 5. This reflects the tendency of the GPs to round blood pressure values, explainable by the wide use of sphygmomanometers. Therefore, the effect on blood pressure was assessed using different criteria. With the generally accepted targets of <140/90 mmHg or a diastolic BP below 90 mmHg, respectively 26 % and 60% of patients were controlled with 150 mg irbesartan qd. In a more recent survey, in which physicians had the opportunity to use the higher dose of 300 mg or the combinations of irbesartan 150 or 300 mg with hydrochlorothiazide 12.5 mg, the percentage of patients with a blood pressure below 140/90 mmHg increases to more than 60% [28]. Looking at sub-populations, older patients and pre-treated patients had more problems in reaching the therapeutic target chosen for analysis (≤ 140/90 mmHg). The same was true for patients with more cardiovascular risk factors, but not for people with associated clinical conditions – the highest risk factor according to the WHO 1999 guidelines, JNC-7 and ESH-ESC 2003 guidelines – who, on the contrary, reached the target more easily [8,9,29]. This observation further emphasize the known difficulty to achieve a good control of systolic blood pressure particularly in elderly patients with isolated systolic hypertension.

In post-marketing surveys in real life settings, the evaluation of safety is of great importance. More than 90% of patients tolerated the treatment well or very well according to their GPs and adverse events were reported by only 8% of them. Serious adverse events possibly or probably related to irbesartan occurred in less than ten cases. The patients with more risk factors tolerated irbesartan equally well, despite taking significantly more drugs. The most frequent adverse events were dizziness, nausea, headache, dyspepsia and diarrhea, and occurred at the rate described in the Swiss prescribing information. These data therefore confirm the excellent tolerability profile of angiotensin II receptor antagonists reported in clinical trials [30].

The main observation of the present survey is the assessment of the factors determining long-term persistence with the irbesartan treatment in our population. Out of the 4639 patients with complete follow-up data, 82.5% continued to take irbesartan for more than 4 months. When evaluating factors affecting persistence some interesting observations were made. The first and expected ones are that a good tolerability profile and the achievement of a rapid blood pressure control are positively associated with the long-term persistence with therapy. This finding would therefore encourage the use of well-tolerated antihypertensive drugs such as angiotensin II receptor antagonists at high doses in order to obtain a rapid control of blood pressure. It may also favor the use of fixed dose combinations as first line treatment since these combinations are associated with a low side-effect profile and an improved efficacy [31].

A consistent majority of the study population (61.5%) entered the trial after a failed experience with other antihypertensive drugs. Irbesartan proved to be the drug inducing more persistence in a comparative study with newly-diagnosed patients [19]; therefore, it was also interesting to appraise the persistence rate in pre-treated patients. To our surprise, patients who switched from a fixed combination treatment tended to discontinue irbesartan more often. On the contrary, patients who abandoned the previous treatment because of cough (a class side effect of ACE-Inhibitors), tended to stick more to irbesartan. Moreover, late changes in treatment schedule had a negative impact on persistence whereas early changes in treatment had a rather favorable impact on persistence. These negative influences on persistence are probably linked to the increased complexity of the treatment schedule which is known to impair compliance as well as persistence. Indeed, several previous studies have demonstrated that drug adherence decreases in proportion with the complexity of the drug regimen [32,33].

In this survey, an attempt was made to obtain information on drug adherence using simplified questionnaires addressed to the patients and physicians. Unfortunately, only a small proportion of these questionnaires were filled by the participants. There is therefore a high probability of bias towards highly compliant patients. Moreover, questionnaires are known to overestimate drug adherence. Hence it is not surprising that good compliance with the dosing schedule was reported by more than 90% of the patients after about one year treatment, meaning that this fraction of the patients reported to have taken irbesartan at least 6 times a week in the preceding 3–4 weeks. Yet, the results of more than 1000 questionnaires confirm previous observations such as the lower compliance in men and the absence of effect of age. Indeed, Degoulet at al have also reported thatmale sex is a variable significantly associated with an increased dropout rate in hypertensive patients attending a hypertension clinic [34]. Of interest is the observation that patients with a low cardiovascular risk appear to have a lower compliance whereas those presenting with an isolated systolic hypertension have a higher compliance. These findings may be related to the patients' perception of their disease and their degree of concern. Thus, patients with a low cardiovascular risk may be less motivated to take their medications whereas patients with high systolic blood pressure may be particularly concerned by their hypertension. In line with this finding, we have found recently that epileptic patients well controlled under treatment are less compliant than those experiencing repeated seizures despite treatment [35].

In conclusion, this survey confirms that irbesartan is a safe and effective antihypertensive drug in clinical practice. A high persistence with irbesartan was found in this program which is likely related to the consistent reductions in blood pressure and the good tolerability profile. This post-marketing survey has also tend to confirm in a large population of patients that achieving an early control of blood pressure may be a positive predictor of persistence. Based on this observation one could encourage physicians to start the antihypertensive therapy more aggressively using higher doses of well tolerated drugs such as angiotensin II receptor antagonists or fixed low-dose combinations in order to improve blood pressure control as well as long-term persistence.

## Competing Interests

All authors had a consultant agreement with both Bristol Myers Squibb and Sanofi-Synthelabo. These companies supported the study financially and monitored the study.

## Authors' contributions

MB has written the paper, BH, PG and BW have contributed to the conception of the study, to the analysis of the data and to the redaction of the paper.

## Pre-publication history

The pre-publication history for this paper can be accessed here:



## References

[B1] Cohn JN, Tognoni G (2001). A randomized trial of the angiotensin-receptor blocker valsartan in chronic heart failure. N Engl J Med.

[B2] Pfeffer MA, Swedberg K, Granger CB, Held P, McMurray JJ, Michelson EL, Olofsson B, Ostergren J, Yusuf S, Pocock S (2003). CHARM Investigators and Committees. Effects of candesartan on mortality and morbidity in patients with chronic heart failure: the CHARM-Overall programme. Lancet.

[B3] Lindholm LH, Ibsen H, Dahlof B, Devereux RB, Beevers G, de Faire U, Fyhrquist F, Julius S, Kjeldsen SE, Kristiansson K, Lederballe-Pedersen O, Nieminen MS, Omvik P, Oparil S, Wedel H, Aurup P, Edelman J, Snapinn S, LIFE Study Group (2002). Cardiovascular morbidity and mortality in patients with diabetes in the Losartan Intervention For Endpoint reduction in hypertension study (LIFE): a randomised trial against atenolol. Lancet.

[B4] Parving HH, Lehnert H, Brochner-Mortensen J, Gomis R, Andersen S, Arner P (2001). The effect of irbesartan on the development of diabetic nephropathy in patients with type 2 diabetes. N Engl J Med.

[B5] Lewis EJ, Hunsicker LG, Clarke WR, Berl T, Pohl MA, Lewis JB, Ritz E, Atkins RC, Rohde R, Raz I (2001). Collaborative Study Group. Renoprotective effect of the angiotensin-receptor antagonist irbesartan in patients with nephropathy due to type 2 diabetes. N Engl J Med.

[B6] Brenner BM, Cooper ME, de Zeeuw D, Keane WF, Mitch WE, Parving HH, Remuzzi G, Snapinn SM, Zhang Z, Shahinfar S (2001). RENAAL Study Investigators. Effects of losartan on renal and cardiovascular outcomes in patients with type 2 diabetes and nephropathy. N Engl J Med.

[B7] Pfeffer MA, McMurray JJ, Velazquez EJ, Rouleau JL, Kober L, Maggioni AP, Solomon SD, Swedberg K, Van de Werf F, White H, Leimberger JD, Henis M, Edwards S, Zelenkofske S, Sellers MA, Califf RM (2003). Valsartan, captopril, or both in myocardial infarction complicated by heart failure, left ventriculardysfunction, or both. N Engl J Med.

[B8] Chobanian AV, Bakris GL, Black HR, Cushman WC, Green LA, Izzo JL, Jones DW, Materson BJ, Oparil S, Wright JT, Roccella EJ (2003). National Heart, Lung, and Blood Institute Joint National Committee on Prevention, Detection, Evaluation, and Treatment of High Blood Pressure; National High Blood Pressure Education Program Coordinating Committee. The Seventh Report of the Joint National Committee on Prevention, Detection, Evaluation, and Treatment of High Blood Pressure: the JNC 7 report. JAMA.

[B9] Guidelines Committee (2003). European Society of Hypertension-European Society of Cardiology guidelines for the management of arterial hypertension. J Hypertens.

[B10] Burt VL, Cutler JA, Higgins M, Horan MJ, Labarthe D, Whelton P, Brown C, Roccella EJ (1995). Trends in the prevalence, awareness, treatment, and control of hypertension in the adult US population. Data from the health examination surveys, 1960 to 1991. Hypertension.

[B11] Gasse C, Hense HW, Stieber J, Doring A, Liese AD, Keil U (2001). Assessing hypertension management in the community: trends of prevalence, detection, treatment, and control of hypertension in the MONICA Project, Augsburg 1984–1995. J Hum Hypertens.

[B12] Marques-Vidal P, Tuomilehto J (1997). Hypertension awareness, treatment and control in the community: is the 'rule of halves' still valid?. J Hum Hypertens.

[B13] Chamontin B, Poggi L, Lang T, Menard J, Chevalier H, Gallois H, Cremier O (1998). Prevalence, treatment, and control of hypertension in the French population: data from a survey on high blood pressure in general practice, 1994. Am J Hypertens.

[B14] Colhoun HM, Dong W, Poulter NR (1998). Blood pressure screening, management and control in England: results from the health survey for England 1994. J Hypertens.

[B15] Jones JK, Gorkin L, Lian JF, Staffa JA, Fletcher AP (1995). Discontinuation of and changes in treatment after start of new courses of antihypertensive drugs: a study of a United Kingdom population. BMJ.

[B16] Caro JJ, Speckman JL, Salas M, Raggio G, Jackson JD (1999). Effect of initial drug choice on persistence with antihypertensive therapy: the importance ofactual practice data. CMAJ.

[B17] Marentette MA, Gerth WC, Billings DK, Zarnke KB (2002). Antihypertensive persistence and drug class. Can J Cardiol.

[B18] Degli Esposti E, Sturani A, Di Martino M, Falasca P, Novi MV, Baio G, Buda S, Volpe M (2002). Long-term persistence with antihypertensive drugs in new patients. J Hum Hypertens.

[B19] Hasford J, Mimran A, Simons WR (2002). A population-based European cohort study of persistence in newly diagnosed hypertensive patients. J Hum Hypertens.

[B20] Larochelle P, Flack JM, Marbury TC, Sareli P, Krieger EM, Reeves RA (1997). Effects and tolerability of irbesartan versus enalapril in patients with severe Hypertension. Am J Cardiol.

[B21] Kassler-Taub K, Littlejohn T, Elliott W, Ruddy T, Adler E (1998). Comparative efficacy of two angiotensin II receptor antagonists, irbesartan and losartan in mild-to-moderate hypertension. Am J Hypertens.

[B22] Mancia G, Korlipara K, van Rossum P, Villa G, Silvert B (2002). An ambulatory blood pressure monitoring study of the comparative antihypertensive efficacy of two angiotensin II receptor antagonists, irbesartan and valsartan. Blood Press Monit.

[B23] Man in't Veld AJ (1997). Clinical overview of irbesartan: expanding the therapeutic window in hypertension. J Hypertens Suppl.

[B24] (2003). Arzneimittel-Kompendium der Schweiz Basel: Documed.

[B25] Schmidt J, Kraul H (1999). Clinical experience with spirapril in human hypertension. J Cardiovasc Pharmacol.

[B26] Kaplan NM (1996). The CARE Study: a postmarketing evaluation of ramipril in 11,100 patients. The Clinical Altace Real-World Efficacy (CARE) Investigators. Clin Ther.

[B27] Speirs C, Wagniart F, Poggi L (1998). Perindopril postmarketing surveillance: a 12 month study in 47,351 hypertensive patients. Br J Clin Pharmacol.

[B28] Ferrari P, Hess L, Pechere-Bertschi A, Muggli F, Burnier M (2004). Reasons for not intensifying antihypertensive treatment (RIAT): a primary care antihypertensive intervention study. Journal of Hypertens.

[B29] (1999). Guidelines Subcommittee. 1999 World Health Organization-International Society of Hypertension Guidelines for the Management of Hypertension. Journal of Hypertens.

[B30] Mazzolai L, Burnier M (1999). Comparative safety and tolerability of angiotensin II receptor antagonists. Drug Safety.

[B31] Waeber B (2003). Very-low-dose combination: a first-line choice for the treatment of hypertension?. Journal of Hypertens.

[B32] Ruud P (1995). Clinicians and patients with hypertension: unsettled issuesabout compliance. American Heart Journal.

[B33] Wuerzner K, Hasler C, Burnier M (2003). Difficult blood pressure control: watch out for non compliance. Nephrol Dial Transplant.

[B34] Degoulet P, Ménard J, Vu HA, Golmard JL, Devries C, Chatelier G, Plouin PF (1983). Factors predictive of attendance at clinic and blood pressure control in hypertensive patients. BMJ.

[B35] Schneider MP, Desplands PA, Buclin Th, Burnier M (2003). Evaluation of online telemonitoring of drug adherence: a pilot randomized, controlled study in patients with epilepsy. J Inform Techn Healthcare.

